# Supplementary material for: Mitochondrial Dysfunction: A Common Hallmark Underlying Comorbidity between sIBM and Other Degenerative and Age-Related Diseases
Source: J Clin Med. 2020 May 13;9(5):1446. doi: 10.3390/jcm9051446 (PMC7290779; doi:10.3390/jcm9051446)
Supplement: Supplementary file 1 [file jcm-09-01446-s001.pdf]

Supplementary Materials:

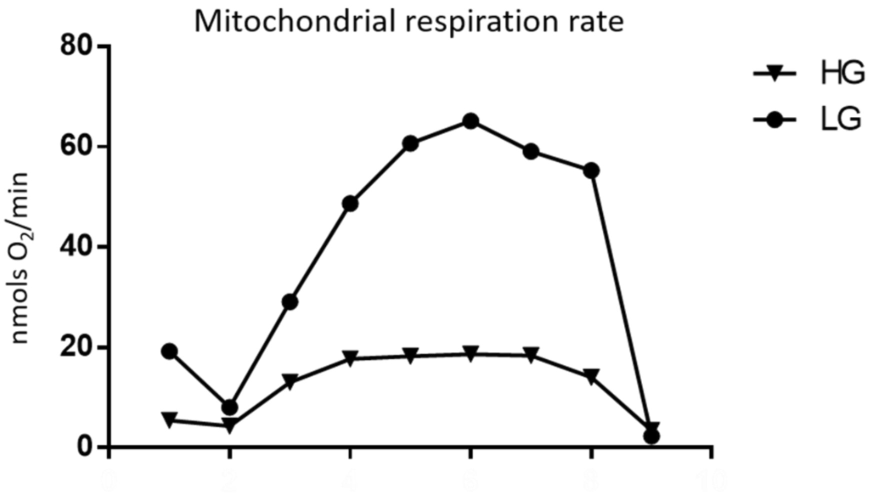

Figure S1. Mitochondrial respiratory activation of control fibroblasts when glucose levels are reduced from HG to LG (HG: high glucose media and LG: low glucose media).
